# A High-Temperature, Low-Noise Readout ASIC for MEMS-Based Accelerometers

**DOI:** 10.3390/s20010241

**Published:** 2019-12-31

**Authors:** Min Qi, An-qiang Guo, Dong-hai Qiao

**Affiliations:** 1 Institute of Acoustics, Chinese Academy of Sciences, Beijing 100190, China; guoanqiang@mail.ioa.ac.cn (A.-q.G.); qiaod@mail.ioa.ac.cn (D.-h.Q.); 2 University of Chinese Academy of Sciences, Beijing 100049, China

**Keywords:** MEMS accelerometers, interface ASIC, high-temperature, low-noise

## Abstract

This paper presents the development and measurement results of a complementary metal oxide semiconductor (CMOS) readout application-specific integrated circuit (ASIC) for bulk-silicon microelectromechanical system (MEMS) accelerometers. The proposed ASIC converts the capacitance difference of the MEMS sensor into an analog voltage signal and outputs the analog signal with a buffer. The ASIC includes a switched-capacitor analog front-end (AFE) circuit, a low-noise voltage reference generator, and a multi-phase clock generator. The correlated double sampling technique was used in the AFE circuits to minimize the low-frequency noise of the ASIC. A programmable capacitor array was implemented to compensate for the capacitance offset of the MEMS sensor. The ASIC was developed with a 0.18 μm CMOS process. The test results show that the output noise floor of the low-noise amplifier was −150 dBV/√Hz at 100 Hz and 175 °C, and the sensitivity of the AFE was 750 mV/pF at 175 °C. The output noise floor of the voltage reference at 175 °C was −133 dBV/√Hz at 10 Hz and −152 dBV/√Hz at 100 Hz.

## 1. Introduction

High-precision accelerometers are widely used in many applications. They play a vital role as a bridge between mechanical vibrations and electrical signals. In some industrial applications, the accelerometers need to work at high temperatures, such as in a structural monitoring system, in a car engine vibration test, or in the downhole equipment in oil and gas production. In such applications, the accelerometers are directly exposed to high-temperature environments ranging from 150 °C to 250 °C. Even with cooling systems, electronic circuits are required to work at 175 °C. Such high-temperature operational environments place a stringent requirement on the readout electronics because the accelerometer’s performance degrades at high temperatures, but the noise must be carefully controlled in precision applications.

Currently, accelerometers are implemented based on a variety of technologies, including piezoelectricity, fiber-optic, molecular electronics transfer (MET), and microelectromechanical system (MEMS). MET accelerometers usually use a liquid electrolyte as their inertial mass [1,2]. They do not contain any precision mechanical parts or springs and are relatively simple to manufacture. The lack of temperature and long-term stability, non-identical amplitude responses prevented them from wide use despite the low cost and acceptable sensitivity. Fiber-optic sensors exhibit unique capabilities in small Spaces, in environments of high electromagnetic interference and high temperature [3]. The output signal of the fiber-optic sensor will be affected by light source fluctuation, fiber transmission loss change, detector aging, and other factors. Moreover, the practicality of the fiber-optic sensor is still to be developed, and its production cost is quite expensive. Piezoelectric accelerometers can operate at temperatures exceeding 250 °C [4]. A charge amplifying circuit for high-temperature piezoelectric accelerometers is presented in [5]. However, piezoelectric accelerometers have limited applications due to their disadvantages of low sensitivity and resolution. Compared to the accelerometers based on the above-mentioned technologies, MEMS capacitive accelerometers demonstrate higher resolution, better linearity [6], integrability with complementary metal oxide semiconductor (CMOS) integrated circuit technology, and reasonable cost for manufacture.

Though many commercial MEMS accelerometers are readily available, the majority of these devices cannot operate in high-temperature environments due to the degradation in the performance of their integrated electronics. An open-loop high-temperature MEMS accelerometer is described in [7], whose readout application-specific integrated circuit (ASIC) is designed to be able to operate in the temperature range of −55 °C to 175 °C. However, the open-loop implementation limits the applications where high linearity is required.

In this paper, we present a high-temperature, low-noise readout ASIC for a MEMS capacitive accelerometer with a bulk-silicon sandwich structure MEMS sensor. The readout ASIC is designed for both open-loop and closed-loop applications. The ASIC features low-noise operation at high temperatures of up to 175 °C.

This paper is organized as follows. Section 2 introduces the structure of the proposed ASIC and depicts the accelerometer employing the ASIC. Section 3 describes details of the circuits design, and Section 4 illustrates the results of the measurement. Finally, the conclusion of this study is presented in Section 5.

## 2. Structure of the Accelerometer Based on the Proposed ASIC

**Figure 1:**
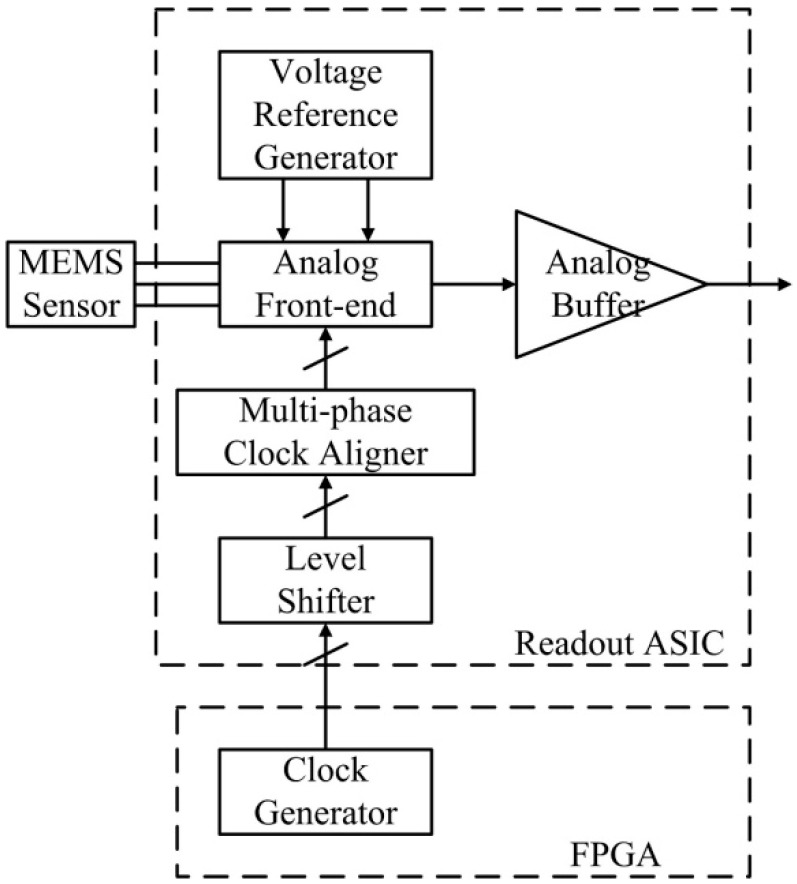
Structure of the open-loop accelerometer with the proposed readout ASIC.

The readout ASIC is designed to interface with a bulk capacitive MEMS sensor in an open-loop application, as shown in Figure 1. The open-loop MEMS accelerometer consists of a differential capacitive MEMS sensor and the proposed readout ASIC. In the ASIC, an analog front-end (AFE) interface with the MEMS sensor, which converts the difference in capacitance resulting from the input acceleration into a voltage signal. An analog buffer provides the driving capability for the analog voltage signal. The ASIC integrates a voltage reference generator that provides a low-noise voltage reference for the AFE. A multi-phase clock aligner is required to adjust the incoming off-chip clocks for the AFE. The off-chip clock generator can be implemented with a field-programmable gate array (FPGA) or microcontroller. Precision clock alignment on board is not required because the clocks are aligned in the ASIC.

**Figure 2:**
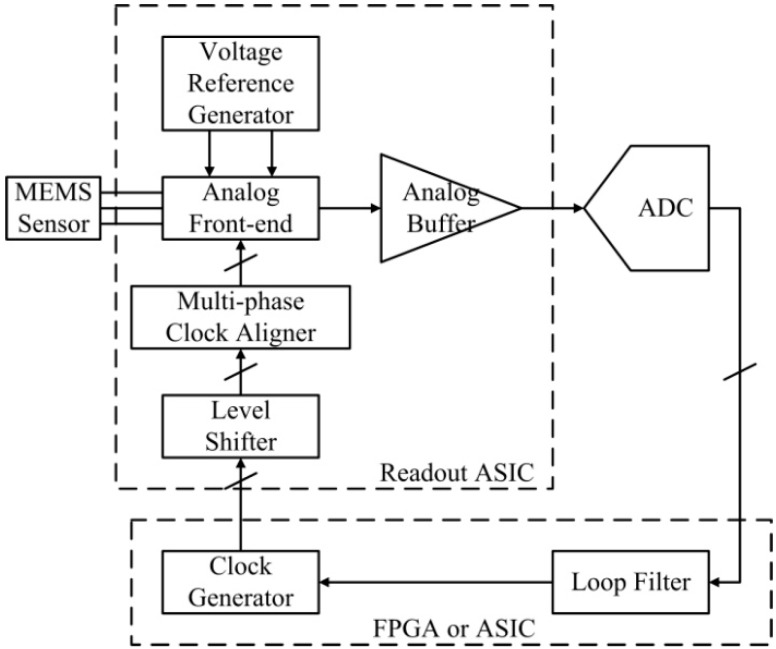
Structure of the closed-loop accelerometer with the proposed readout ASIC.

Besides the basic functionality for open-loop operation, the ASIC includes extra circuits that make it possible to upgrade the open-loop accelerometer to a closed-loop accelerometer with the use of additional off-chip circuits. In the closed-loop accelerometer, an analog-to-digital converter (ADC), following the proposed ASIC, converts the analog signals representing the acceleration into digital signals. Then, a digital loop filter plays the signal using algorithms and sends feedback to the ASIC through the clock generator, as illustrated in Figure 2. The digital loop filter and the clock generator can be implemented with an FPGA or an ASIC.

Taking advantage of the use of high-voltage devices in the process, the analog building blocks in the ASIC work at a dual supply of ±5 V to accommodate a high voltage for the static force feedback from the closed-loop operation.

**Figure 3:**
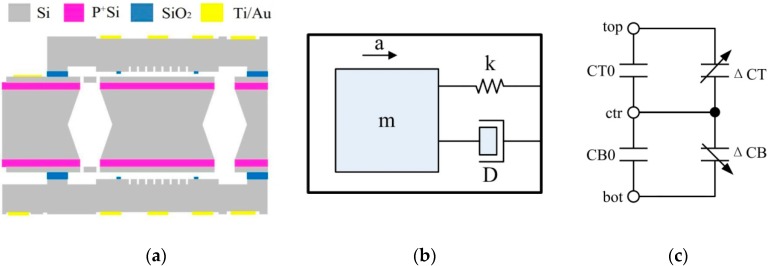
(**a**) Structure of the sandwich-type accelerometer; (**b**) 2nd order model of the mechanical device; (**c**) Equivalent circuit of the mechanical device.

The sensor, paired with the proposed ASIC, is a sandwich-type capacitive MEMS sensor. Compared with a comb-drive type sensor, a sandwich-type has many advantages, including larger proof mass, larger capacitance, and higher resolution. A sandwich-type capacitive MEMS sensor consists of two fixed plates and a movable proof mass, as shown in Figure 3a [8]. Figure 3b is the 2nd order model of the mechanical structure. The movement of the proof mass converts the acceleration into a variation in capacitance. The Brownian noise equivalent acceleration (BNEA) of the MEMS sensor in Figure 3 can be expressed as in Equation (1), which is the main noise source of the sensor.

(1) $$\mathrm{BNEA}=\frac{1}{g}\frac{\sqrt{{4k}_{B}TD}}{m}\left[\frac{g}{\sqrt{Hz}}\right],$$

where k_B_ is the Boltzmann constant (1.38 × 10^−23^ J/K), T is the temperature in Kelvin, *D* is the damping factor, m is the mass of the proof mass, and *g* is the gravitational acceleration (9.8 m/s^2^). In this case, the damping factor is 2.4 × 10^−3^ N·s/m, and the mass of the proof mass is 2.6 × 10^−5^ kg. The BNEA of the sensor is 25 ng/√Hz at T = 300 K and 30 ng/√Hz at *T* = 448 K, which are much lower than the noise level of the readout circuit.

The equivalent circuit of a sandwich-type accelerometer is shown in Figure 3b. *C_T_*_0_ and Δ*C_T_* are the initial capacitor and capacitance variation between the top plate and the center plate, respectively. *C_B_*_0_ and Δ*C_B_* are the initial capacitor and capacitance variation between the bottom plate and the center plate, respectively. In total, *C_T_* = *C_T_*_0_ + Δ*C_T_* and *C_B_* = *C_B_*_0_ + Δ*C_B_*. When the accelerometer has zero acceleration input, the center plate is precisely in the middle and *C_T_* = *C_B_*. When the center plate moves due to acceleration, the difference between *C_T_* and *C_B_* is:

(2) $$C_{T}-C_{B}=\varepsilon_{0}\varepsilon \left(\frac{1}{d_{0}-x}-\frac{1}{d_{0}+x}\right)\approx \frac{2\varepsilon_{0}\varepsilon Ax}{d_{0}^{2}}\left(x\ll d_{0}\right),$$

where *x* is the displacement of the center plate, *d*_0_ is the initial gap between the top plate and the center plate, *ε*_0_ is the vacuum permittivity, *ε* is the relative dielectric constant, and A is the area of the plate. Equation (2) reveals that when *x* << *d*_0_, the displacement of the proof mass is proportional to *C_T_* − *C_B_* [8].

## 3. Design of the ASIC

### 3.1. Analog Front-End

One of the functions of the analog front-end is detecting the capacitance variation of the capacitive sensor, and converting the capacitive signal to a voltage signal. In-band noise is a significant specification of the capacitance to voltage conversion. In order to minimize the in-band noise, one or more noise reduction techniques must be used. The chopper-stabilization (CHS) technique is usually used to reduce the noise of a readout circuit [9]. However, CHS matches well with a differential-difference amplifier, which is not suitable for out circuits due to the added complexity. The analog front-end in this paper uses the correlated double sampling (CDS) technique [10] for low-frequency noise reduction.

**Figure 4:**
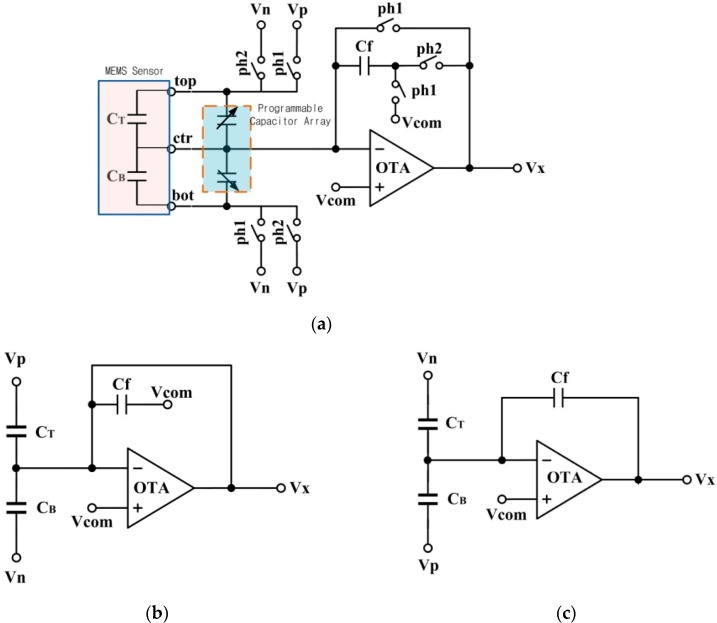
(**a**) Structure of the MEMS readout circuit with CDS technique; (**b**) Equivalent circuit of *PH*1 stage; (**c**) Equivalent circuit of *PH*2 stage.

Figure 4a shows the simplified schematic of the analog front-end for the capacitance-to-voltage conversion. The conversion includes two operational phases for the CDS operation, defined as *PH*1 and *PH*2, shown in Figure 4b,c. In the PH1 stage, the top plate of the sensor is connected to the positive reference voltage (*V_p_*), and the bottom plate is connected to the negative reference voltage (*V_n_*). The feedback capacitor (*C_f_*) is connected between the reversed input of the operational transconductance amplifier (OTA) and the common-mode voltage (*V_com_*). The charges on the three capacitors are *Q_T_* (*PH*1) = (*Vp* − *Vcom*) *C_T_*, *Q_B_* (*PH*1) = (*V_n_* − *V_com_*) *C_B_*, and *Q_f_* (*PH*1) = 0.

In the *PH*2 stage, the top plate of the sensor is connected with *V_n_* and the bottom plate is connected with *V_p_*. The charges on *C_T_* and *C_B_* transfer to *C_f_*. The charges on the three capacitors are *Q_T_* (*PH*2) = (*V_n_* − *V_com_*) *C_T_*, *Q_B_* (*PH*2) = (*V_p_* − *V_com_*) *C_B_*, and *Q_f_* (*PH*2) = (*V_x_* − *V_com_*) *C_f_*. Equation (3) is derived from the law of conservation of charge:

(3) $$Q_{T}\left(PH1\right)+Q_{B}\left(PH1\right)+Q_{f}\left(PH1\right)=Q_{T}\left(PH2\right)+Q_{B}\left(PH2\right)+Q_{f}\left(PH2\right).$$

The output voltage of the AFE is:

(4) $$V_{x}=\frac{\left(V_{p}-V_{n}\right)\left(C_{T}-C_{B}\right)}{C_{f}}+V_{com}.$$

Another function of the AFE is performing static force feedback, which is used for the closed-loop accelerometer. Additional switches are implemented in the AFE. When performing the static force feedback, a voltage reference is applied to one of the capacitors in the MEMS sensor. Then, when the proof mass is moved, the capacitance is changed by the static force formed between the two plates. The feedback direction will determine which capacitor is chosen to apply the static force.

### 3.2. Operational Transconductance Amplifier

**Figure 5:**
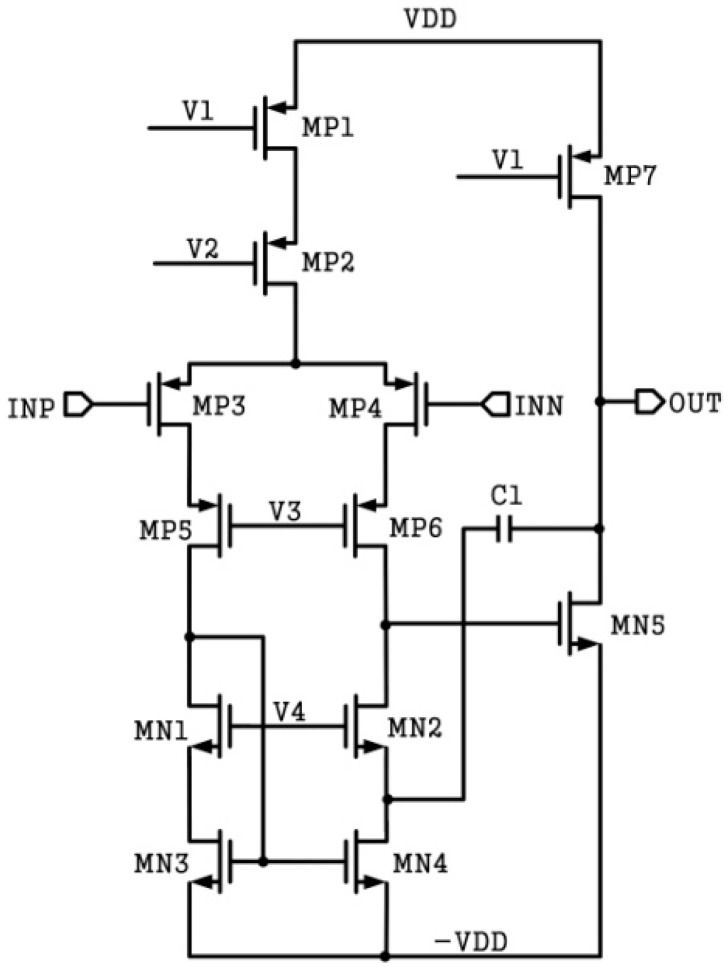
OTA schematic of analog front-end.

The OTA of the AFE requires both high gain and low noise. In this paper, a two-stage operational amplifier with Miller compensation is used. The schematic of the OTA is shown in Figure 5. The cascode amplifiers, whose gain can meet the requirements, include the telescopic type and the folded type. In many applications, the folded cascode structure is adopted for a larger output swing [8,11]. Compared with the folded type, the telescopic type has higher speeds, lower power consumption, and lower noise [12]. In this design, a telescopic structure is used in the first stage to provide the necessary gain while maintaining low noise. The second stage is a common source amplifier, which has a large output swing.

In order to reduce current leakage, which increases with temperature, various compensation measures have been adopted for different designs [13,14]. However, the effectiveness of these methods is very limited. A high-temperature process is employed in this case, which can minimize current leakage at high temperatures through a special doping process and a complex device structure. Owing to the special model of the high-temperature process, the threshold voltage of a high-voltage metal-oxide-semiconductor field-effect transistor (MOSFET) is much higher than the general device. In order to reduce the influence of the high threshold voltage, transistor MP3, MP4, MN3, and MN4 in Figure 5 use conventional low-voltage devices.

### 3.3. Voltage Reference Generator

**Figure 6:**
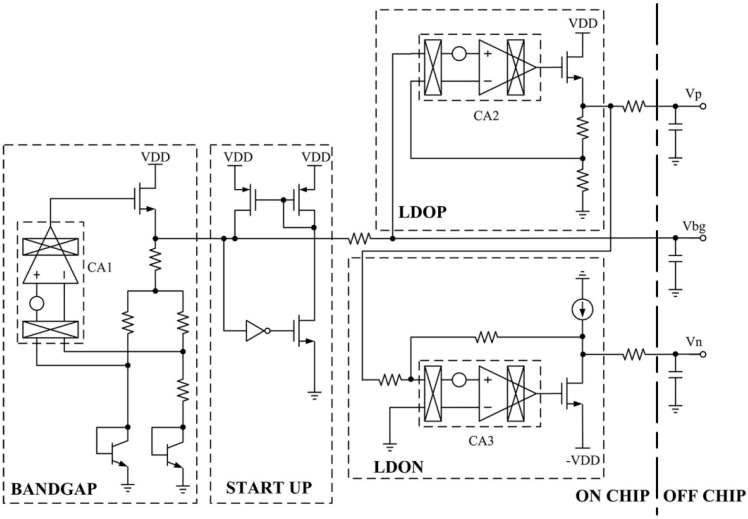
Block diagram of the voltage reference generator.

The voltage references *V_n_* and *V_p_* in Figure 4 are generated by the circuit in Figure 6. The noise performance of the voltage reference directly affects the noise performance of the analog front-end. There are two basic techniques that are used to reduce the offset and low-frequency noise of operational amplifiers, namely the autozero (AZ) and CHS techniques [9]. A clear distinction is made between AZ, which is a sampling technique, and CHS, which is a modulation technique, mainly with respect to their effect on the amplifier broadband noise. The CHS technique is employed in this paper to reduce low-frequency noise, effectively. In Figure 6, CA1, CA2, and CA3 are CHS operational amplifiers [15]. The BANDGAP module is used to generate the bandgap voltage (*V_bg_*) [16]. The LDOP and LDON modules are used to generate *V_p_* and *V_n_*, respectively.

## 4. Measurement Results

**Figure 7:**
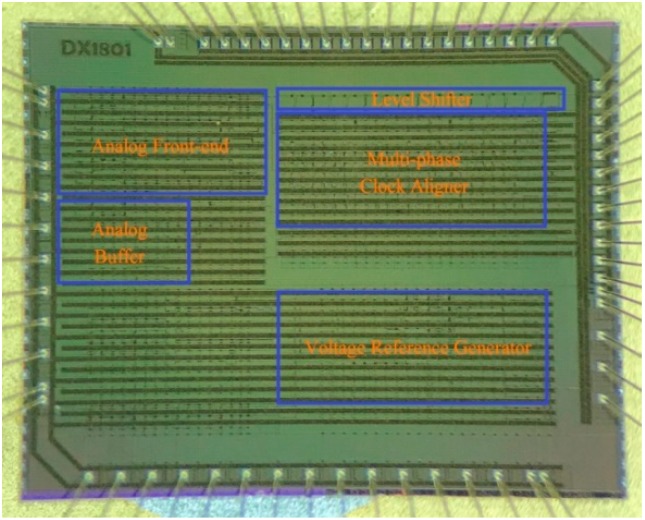
Micro-photo of the readout ASIC.

The proposed readout ASIC is fabricated with a 0.18 μm high-temperature CMOS process. The size of the die is 3.8 × 3.0 mm^2^. Figure 7 shows a micro-photo of the die.

**Figure 8:**
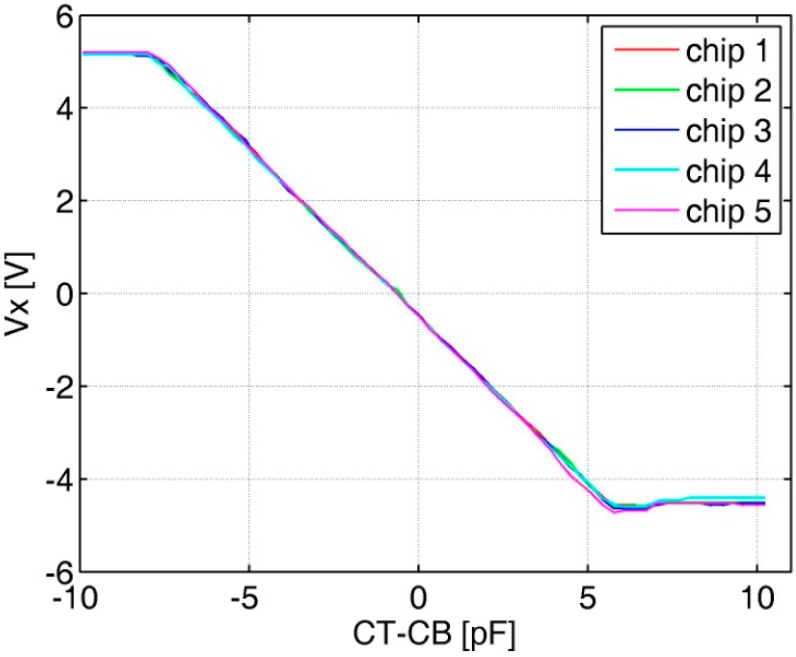
Analog front-end test results with the on-chip programmable capacitor.

The functionality of the AFE is tested with the assistance of an on-chip programmable capacitor array. Because the measured *C_T_* or *C_B_* of the typical sensor is equal to about 200 p, so two 200 pF capacitors are used to simulate the differential capacitance from the MEMS sensor, while the on-chip capacitor array is scanned with a step of 160 fF during the test. Figure 8 shows the test results of five prototypes. An average capacitance-to-voltage gain measures 750 mV/pF with good linearity.

**Figure 9:**
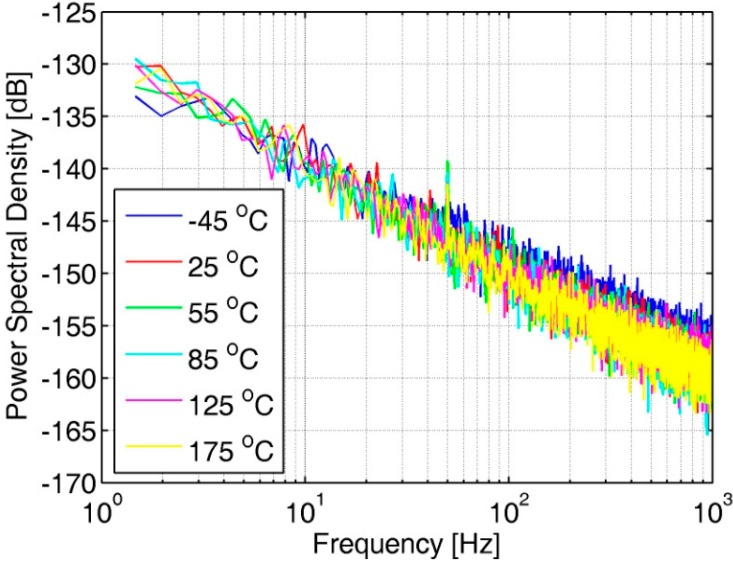
Noise PSD of the OTA in the analog front-end at various temperature points.

A copy of the OTA in the AFE is placed on the chip and tested. Figure 9 shows the measured power spectral density (PSD) of the OTA copy at various temperature points. In the low-frequency band below 1 kHz, the noise of the amplifier is mainly flicker noise, also known as 1/f noise. No degradation of the OTA noise is observed at high temperatures because the OTA performance is designed for high temperatures.

**Figure 10:**
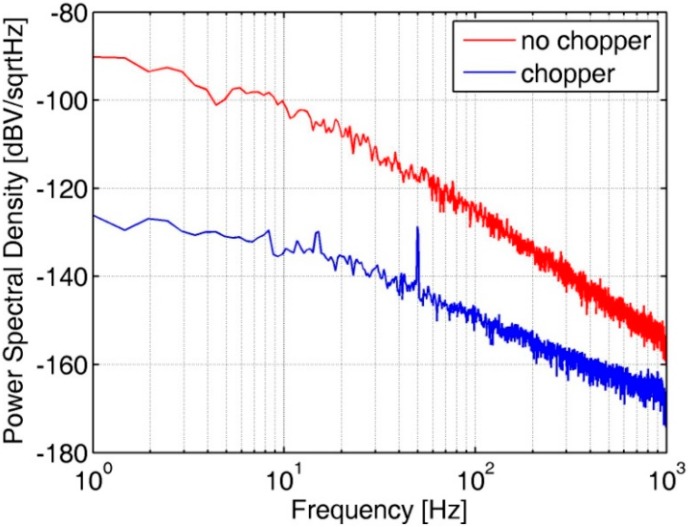
Noise PSD of the bandgap voltage reference at 175 °C.

The noise PSD of the bandgap voltage, which is the source of the voltage reference, is measured. Figure 10 shows the noise PSD of the bandgap voltage with CHS on and off at 175 °C. Low-frequency noise is clearly suppressed when the CHS is on.

A summary of the measurement noise results for the proposed bandgap along with a comparison of performance between the other five references is shown in Table 1. It can be seen that this work exhibits good noise performance at a wide temperature range.

**Figure 11:**
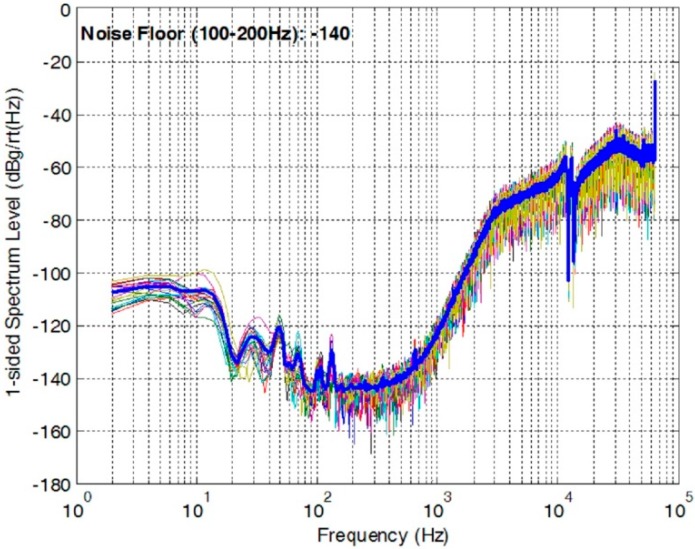
Noise PSD of a closed-loop accelerometer at room temperature.

The OTA and the bandgap reference are top-two significant noise contributors in a precision MEMS accelerometer with the similar structures shown in Figure 1 and Figure 2, which have been validated through extensive behavior level simulation. A closed-loop accelerometer has been implemented with a similar structure in Figure 2, which measured a noise-floor of −140 dBg/√Hz, as shown in Figure 11. The noise below 100 Hz was introduced by weak vibration at low frequency. Though this closed-loop accelerometer was designed for room temperature operation, we extracted the noise performance of the OTA and the bandgap and used them as the goals for the high-temperature designs in this paper. Table 2 summarizes the measured noise performance for the high-temperature circuits proposed in the paper and the goals of the design extracted from the room-temperature accelerometer.

## 5. Conclusions

This work demonstrates the development and the test of a readout ASIC for MEMS accelerometers. The test results show that the output noise floor of the low-noise amplifier at 175 °C is −150 dBV/√Hz ≅ 100 Hz, and the sensitivity of the analog front-end is 750 mV/pF. The output noise floor of bandgap at 175 °C, adopting the chopper-stabilized technique, is −133 dBV/√Hz ≅ 10 Hz and −152 dBV/√Hz ≅ 100 Hz. It’s concluded that the test results have met the design goals. This work can meet the requirement of certain high-temperature applications. The readout circuits are suitable for both the open-loop accelerometer and closed-loop systems with higher accuracy [22].

## Figures and Tables

**Table 1:** Performance of Bandgaps.

Parameter	[17]	[18]	[19]	[20] ^1^	[21] ^1^	This Work
Temperature range (°C)	−40–125	−40–125	−40–125	0–100	−40–80	−45–175
Process	0.16 µm CMOS	0.35 µm CMOS	0.5 µm BiCMOS ^2^	Bipolar	2.0 µm CMOS	0.18 µm HT CMOS ^3^
Noise (uV/√Hz) ≅ 1 Hz	2	5	0.15	0.166	0.17	0.5092
Noise (uV/√Hz) ≅ 10 Hz	1	1.8	0.08	0.166	0.17	0.2071
Noise (uV/√Hz) ≅ 100 Hz	1	0.5	0.04	0.166	0.17	0.0341

^1^ Simulation Data. ^2^ Bipolar Complementary Metal Oxide Semiconductor. ^3^ High-Temperature Complementary Metal Oxide Semiconductor.

**Table 2:** Results between simulation data and measured data.

Parameter	Simulation	Measurement
Temperature range (°C)	−45–175	−45–175
Sensitivity (mV/pF)	730	750
Noise of OTA (dBV/√Hz)	−140 ≅ 100 Hz	−150 ≅ 100 Hz
Noise of bandgap (dBV/√Hz)	−145 ≅ 100 Hz	−152 ≅ 100 Hz
